# The relationship between tooth size discrepancy and archform classification in orthodontic patients

**DOI:** 10.4317/jced.52208

**Published:** 2015-04-01

**Authors:** Gerard O’Mahony, Declan T. Millett, Michael S. Cronin, Grant T. McIntyre, Mark K. Barry

**Affiliations:** 1 Specialist in Orthodontics, Orthodontic Unit, Oral Health and Development, University Dental School and Hospital, Wilton, Cork, Ireland; 2 Professor, Orthodontic Unit, Oral Health and Development, University Dental School and Hospital Wilton, Cork, Ireland; 3 Lecturer in statistics, school of mathematical sciences, University College Cork; 4 Consultant / Senior Lecturer in Orthodontics, Dundee Dental Hospital, 2 Park Place, Dundee, DD1 4HR, UK; 5 Lecturer, School of Manufacturing Engineering, Dublin Institute of Technology, Dublin, Ireland

## Abstract

**Background:**

To determine the relationship between clinically significant tooth size discrepancies (TSD) and archform classification in orthodontic patients.

**Material and Methods:**

Eighty teeth with artificial white spot lesions were randomly divided into four groups: (A) distilled and deionized water, (B) Nd:YAG laser, (C) CPP-ACP crème, & (D) CPP-ACP plus laser. SMH was measured using Vickers diamond indenter in Vickers Hardness Number (VHN). Two samples of each group were analyzed using scanning electron microscope (SEM). The results were analyzed with the SPSS 17/win.

**Results:**

Reproducibility of the classification of archform was very good (unweighted Kappa statistic of 0.83 with a 95% confidence interval of 0.73, 0.93). There was no statistically significant difference in the distribution of archform type between group 1 and group 2 for the upper (p=0.3305) or lower (p=0.6310) arches.

**Conclusions:**

The presence of a clinically significant TSD and archform classification do not appear to be related.

** Key words:**Tooth Size, Archform, Bolton discrepancy, digital models, polynomial curve, archform classification.

## Introduction

A tooth size discrepancy (TSD) exists when the maxillary and mandibular teeth are not in proportion with each other (1). Anterior TSDs involve the six anterior teeth whereas overall TSDs relate to all teeth excluding second permanent and third molars. Both anterior and overall TSDs are relatively common in patients undergoing orthodontic treatment with a prevalence of 4-11% (2-5) and 17-38% (2,4,6-8), respectively. TSDs may be influenced by malocclusion type, gender or race (9).

The pre-treatment archform is important in orthodontic treatment planning (10). Determined by genetic and environmental factors, it is modulated through the skeletal base and soft tissues (11). Alteration in archform during treatment is generally regarded as potentially unstable (12) and where this occurs, the changes should be assessed and quantified (13).

The most common method of assessing a TSD involves the measurement of the mesio-distal widths of teeth using either calipers (1) or computer software packages (14) with the latter having the advantage of automatic calculation of tooth size ratios. Measurements made from digital study models have been found to be an appropriate alternative to those derived from plaster models (15).

Many different descriptions of the dental archform have been proposed (16). These include the Bonwill-Hawley archform constructed around an equilateral triangle in which the mesio-distal width of the lower six anterior teeth form the arc of a circle, Black’s semi-ellipse, Angle’s parabolic curve, the catenary curve and the Brader tapered catenary curve. The development of morphometrics and the use of computer modeling have led to a variety of attempts to describe the form of the dental arch (17) including the beta function, cubic spline, thin-plate spline, Bezier curves, Euclidean data analysis and polynomial functions (17,18). Because there is no general agreement on how best to describe archform, limited epidemiological evidence exists with regards to the prevalence of differing archform types in relation to malocclusion, gender and race (16,19,20).

Tooth size exhibits a continuous range of variation with a strong inheritance pattern; the genetic contribution to mesiodistal and buccolingual crown diameters is over 80% (21). Cassidy *et al.* (22) investigated the genetic influence on the dental archform in 320 adolescents from 155 sibships seeking orthodontic treatment, and found that arch size and arch shape (length-width ratio) has a modest genetic component. The relationship between the dimensions of the anterior teeth and their respective archforms has only been assessed in one study (23). Among 200 Greek subjects seeking orthodontic treatment, a statistically significant relationship between ‘wide’ and ‘pointed’ maxillary archforms with smaller tooth sizes was identified but this was more marked in male subjects (23). Furthermore, a statistically significant relationship between ‘flat’ maxillary archforms and smaller teeth was found in female subjects (23). Only, however tooth size, as opposed to TSD was assessed in that study (23). The relationship between TSD and archform has, therefore, not been evaluated as yet.

The objective of this study was to determine the relationship between a clinically significant TSD and archform in orthodontic patients. The null hypothesis was no relationship exists between a clinically significant TSD and archform in a cohort of orthodontic patients.

## Material and Methods

From a university orthodontic archive, 240 pre-treatment study models were selected using the following criteria: all permanent teeth erupted in each quadrant (except second and third molars), absence of marked rotations, absence of gingival / periodontal problems, no factors precluding precise measurement of tooth widths (including fractured teeth and restorations), no retained primary teeth, no abnormal dental morphology and subjects of the same ethnic background as determined from case records and no history of orthodontic treatment. These models comprised the first 60 sets of each malocclusion group (Class I, Class II division 1, Class II division 2 and Class III) with 30 males and 30 females in each group (24). Following scanning with an R250 Orthodontic Study Model Scanner (3Shape A/S, Copenhagen, Denmark), OrthoAnalyzerTM (3Shape A/S, Copenhagen, Denmark) was used to evaluate the resulting digital images. Although the Declaration of Helsinki principles were followed, research ethics committee was not required as the digital models were not identifiable. One assessor landmarked the maximal widths at the mesial and distal contacts of all teeth (excluding second and third molars) from the occlusal aspect as per Horton *et al.* (25). Bolton tooth size ratios (overall and anterior) (1) were then automatically calculated. A clinically significant TSD was deemed to exist where a Bolton ratio was more than two standard deviations from the mean (1). Two groups with 30 subjects in each were identified; group 1 consisted of images where a significant TSD was present and group 2 where this was absent.

-Mean archform 

A set of fiducial horizontal and perpendicular lines were recorded on each image in OrthoAnalyzerTM to allow subsequent resizing as necessary. The images were then exported as Paint images (Microsoft, Redmond, California). The following points were recorded on each image as per Felton *et al.* (13) and Noorozi *et al.* (26): the mesio-buccal cusp tips of first molars, the buccal cusp tips of premolars, cusp tips of canines and mid-incisal edge points. The contact between the upper and lower central incisors was used as the x,y zero co-ordinate to allow consistent superimposition of the images (13). The contacts between the first molars and second premolars and between second and first premolars were then recorded for each image. These points were used to draw two horizontal lines between the corresponding contacts on the right and left of each image. A mean of these two lines was then used as the horizontal reference line to allow consistent orientation of the archform. The x and y coordinates for each landmark were then identified and recorded using Mathlab and then imported into Excel (Microsoft, Redmond, California). A spreadsheet was compiled and formulae used to align the arches. The images were resized, oriented to the x,y axis and then rotated before the mean of each landmark point was calculated. A best fit 4th degree polynomial curve (the mean archform) (18) was created through these mean landmarks for upper and lower arches for both groups.

-Archform Type

The archform images were then classified as square, tapering or ovoid as previously described (20,26) using 3M-Unitek archform templates (Monrovia, California) (27). This was done by superimposing the templates on the real size digital images of the study models using a best-fit approach to match the landmark points. The templates were superimposed on the anterior eight contacts from first premolar to first premolar as per Kook *et al.* (20).

-Statistical analysis

To assess intra-observer reliability of landmark identification a 10% random sample of the digital images were re-landmarked six weeks after the initial assessment and differences evaluated using 2-way ANOVA (28). To evaluate the intra-operator error for determining archform type, the 60 archforms were re-classified by the same operator two weeks later. The unweighted Kappa statistic at 95% confidence intervals was used to determine the similarity of the classification on the two occasions. The distribution of archform type in each group for either arch was analyzed using the Fisher test with a 5% level of significance.

## Results

-Reliability

There were no statistically significant errors associated with the measurement of overall tooth size ratios [mean difference=0.004 (SD=0.011)] or anterior tooth size ratios [mean difference=0.0001 (SD=0.014)]. Reproducibility of the classification of archform was very good (unweighted Kappa statistic of 0.83 with a 95% confidence interval of 0.73, 0.93).

-Mean archform 

**Figure 1:**
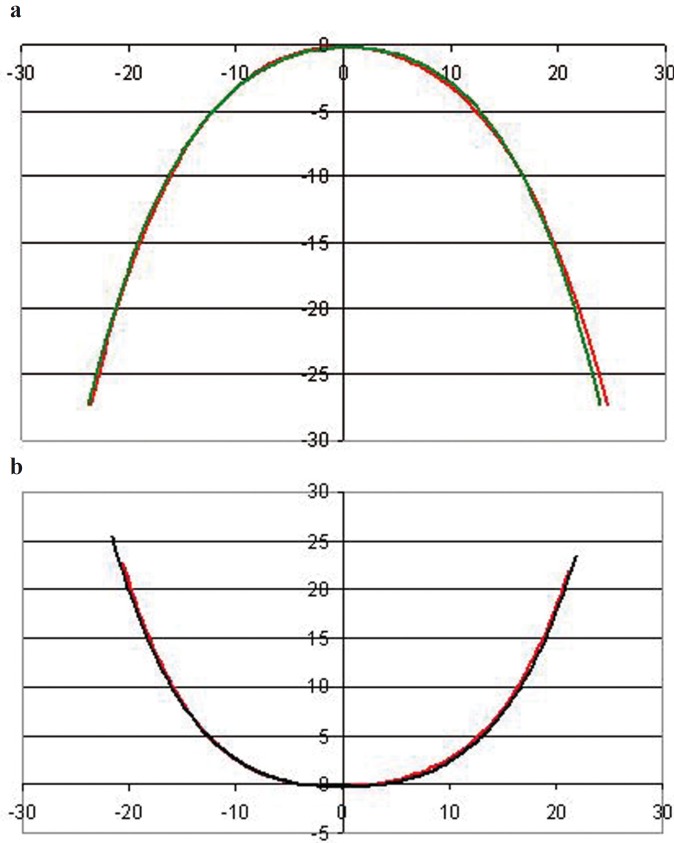
a) The mean upper archform* generated for Group 1 and Group 2†. b) The mean lower archform* generated for Group 1 and Group 2† *The 0,0 point on the x,y axis represents the contact of the central incisors. The x and y axes are measured in millimeters with each line indicating 10 mm along the axis. † Green line represents Group 1 (absence of a clinically significant TSD) and red line represents Group 2 (presence of a clinically significant TSD).

The mean upper and lower archforms for Group 1 and Group 2 are shown in figure 1; with no observable differences between the two groups.

-Archform Type

There were no statistically significant differences in the distribution of archform type between the groups for the upper (p=0.3305) or lower (p=0.6310) arches ( Table 1). Therefore the null hypothesis was supported. The most common archform type found in the upper arch in both groups was tapering. In the lower arch, there was an increase in the number of ovoid archforms when compared to the upper archform groups.

**Table 1:**
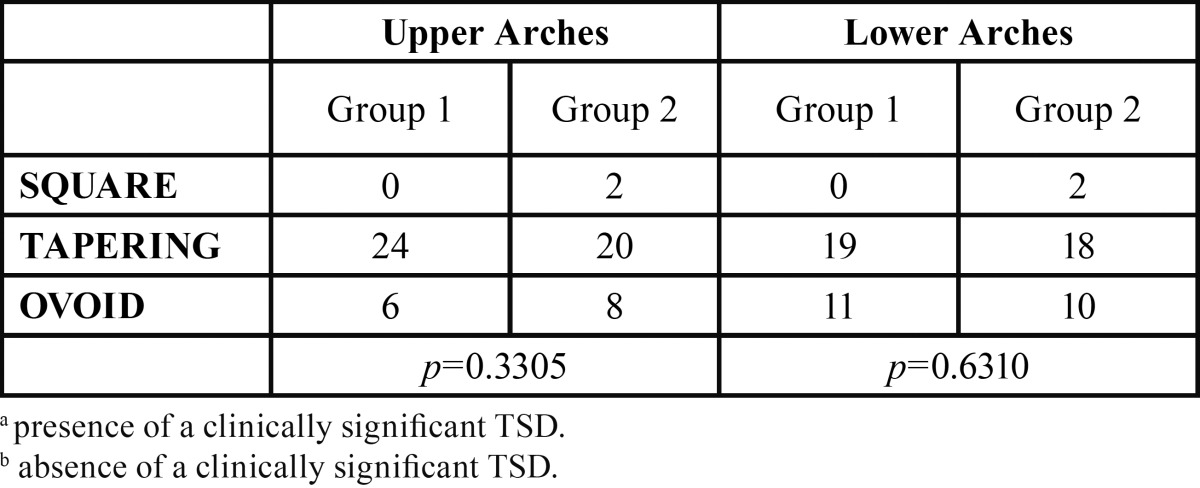
Distribution of archform type in Group 1a and Group 2b.

## Discussion

We found that a clinically significant (overall or anterior) TSD was not associated with type and prevalence of archform in this sample of orthodontic patients. As no other study has assessed the relationship between TSD and archform in orthodontic patients, it is not clear if this may exist in other population groups. Only Haralabakis *et al.* (23) has to date found any relationship between tooth dimensions and arch dimensions in their sample of 200 Greek subjects referred for orthodontic treatment. Two other non-orthodontic investigations have investigated tooth size and arch dimensions (29,30). In their study of 66 mixed subjects (referred patients, undergraduate dental students and dentists), Ng *et al.* (29) found that arch length and circumference were marginally larger in subjects with impacted third molars, particularly in females, although the size of the impacted third molar was not related to the arch dimensions. Sellen *et al.* (30) investigated denture tooth selection and found that there was an insignificant correlation between facial shape, tooth form and archform in 50 dentate undergraduate students. Neither found a relationship between tooth size and arch dimensions and our results are in accordance with these.

Confounders in our study may have arisen from sample heterogeneity. Although similar case types have been used in other investigations of archform (18), severe rotations may adversely affect landmark identification and archform classification. This investigation only assessed patients referred for orthodontic treatment; only on rare occasions are patients referred for treatment to address a TSD and this contribution to bias would be expected to be minimal.

Digital models have not been used to assess archform but are a reliable means of evaluating tooth size (15,28). The polynomial function has been shown to represent three generic archform classification types (square, tapering and ovoid) (20,26,27) and subjective classification of archform has been shown to be highly reliable (26). Using this methodology, the present study confirms the reproducibility of archform classification using digital models. Other studies have used the cusp tips of premolars and molars (13,16) and landmarks taken from the vestibular surface of the teeth (19) whist the use of the maximal widths at the mesial and distal contacts was found to be reliable in our investigation.

With regard to the upper arch, the tapering archform was the most common type in both groups whereas the prevalence of ovoid archforms was greater in the lower. Whilst a relationship between tooth size and archform was not found to exist in this study, clinicians should determine the archform type to be used throughout treatment rather than adjusting the archform when reaching the working archwire stage (13).

The present study found the presence of a clinically significant TSD and archform classification do not appear to be related.
